# Tracing the potential of networks to improve community cancer care: an in-depth single case study

**DOI:** 10.1186/s43058-021-00190-1

**Published:** 2021-08-25

**Authors:** Jennifer L. Ridgeway, Lisa A. Boardman, Joan M. Griffin, Timothy J. Beebe

**Affiliations:** 1 Robert D. and Patricia E. Kern Center for the Science of Health Care Delivery, Mayo Clinic, Rochester, MN 55905 USA; 2 Division of Gastroenterology and Hepatology, Mayo Clinic, Rochester, MN 55905 USA; 3 Division of Health Care Delivery Research and Robert D. and Patricia E. Kern Center for the Science of Health Care Delivery, Mayo Clinic, Rochester, MN 55905 USA; 4 Division of Health Policy and Management, University of Minnesota School of Public Health, Minneapolis, MN 55455 USA

**Keywords:** Networks, Relationship centered care, Care coordination, Community cancer care, Cancer disparities, Knowledge diffusion

## Abstract

**Background:**

Despite overall declines in cancer mortality in the USA over the past three decades, many patients in community settings fail to receive evidence-based cancer care. Networks that link academic medical centers (AMCs) and community providers may reduce disparities by creating access to specialized expertise and care, but research on network effectiveness is mixed. The objective of this study was to identify factors related to whether and how an exemplar AMC network served to provide advice and referral access in community settings.

**Methods:**

An embedded in–depth single case study design was employed to study a network in the Midwest USA that connects a leading cancer specialty AMC with community practices. The embedded case units were a subset of 20 patients with young-onset colorectal cancer or risk-related conditions and the providers involved in their care. The electronic health record (EHR) was reviewed from January 1, 1990, to February 28, 2018. Social network analysis identified care, advice, and referral relationships. Within-case process tracing provided detailed accounts of whether and how the network provided access to expert, evidence-based care or advice in order to identify factors related to network effectiveness.

**Results:**

The network created access to evidence-based advice or care in some but not all case units, and there was variability in whether and how community providers engaged the network, including the path for referrals to the AMC and the way in which advice about an evidence-based approach to care was communicated from AMC specialists to community providers. Factors related to instances when the network functioned as intended included opportunities for both rich and lean communication between community providers and specialists, coordinated referrals, and efficient and adequately utilized documentation systems.

**Conclusions:**

Network existence alone is insufficient to open up access to evidence-based expertise or care for patients in community settings. In-depth understanding of how this network operated provides insight into factors that support or inhibit the potential of networks to minimize disparities in access to evidence-based community cancer care, including both personal and organizational factors.

Contributions to the literature

- Prior research on network effectiveness has focused on discreet evidence-based practices. Case studies of network variability can untangle how and when networks function (un)successfully and thus identify strategies for improvement.
- Networks may minimize community disparities by supporting evidence-informed care close to home and creating referral access as needed. This study’s multi-method approach to analyzing narratives highlighted how provider and patient interactions foster or serve as barriers to these goals.
- Network potential is minimized if they are not activated. Interventions are needed to bolster network adoption among community providers, while balancing the benefits and burdens of increased interactions.

## Background

Cancer statistics in the USA portray significant improvements in survival and mortality in recent decades [1–3], attributed in part to scientific discoveries leading to better prevention, detection, and treatment [4]. Despite these successes, improvements in outcomes are not evenly distributed across the USA patient population [3, 5]. Patients in rural areas are less likely to receive recommended cancer screening, are diagnosed later, and are less likely to receive guideline-concordant cancer treatment than their urban counterparts [6–9]. Treatment at high-volume facilities and comprehensive cancer centers is associated with greater adherence to guideline-concordant and recommended treatment [10–12] and significantly better cancer-related outcomes when compared to unaffiliated and low-volume facilities [13–16]. However, access to these facilities is uneven [17, 18]. Networks that connect community practices to organizations doing research and providing high-volume specialty care may reduce these disparities by addressing barriers providers face to applying research evidence in their practice. These include lack of awareness or lack of time to search for evidence [19–21]. Provider decision-making may also be constrained by factors outside their control, including insurance policies that fail to recognize clinical guidelines [22, 23] or lack of specialty care referral access [24], even when evidence-based guidelines call for them [25]. Networks may offset these barriers by creating administrative links and referral access, as well as access to expert advice. In fact, research has shown that community practices affiliated with a medical school or cancer research network provide better access to evidence-based care than non-affiliated centers [26–28]. This includes better access to treatments and clinical trials [26, 29, 30].

Despite the potential of networks, research suggests some variability in network effectiveness when comparing whether patients are more likely to receive a specific evidence-based practice, such as a recommended treatment or screening innovation, if their facility is part of a research network compared to patients in non-networked facilities [31, 32]. Further research is needed to understand not just whether but how network arrangements lead to evidence-based care for community patients. Furthermore, research is needed that goes beyond the uptake of a single evidence-based practice to understand a twofold view of network benefits: (1) they may provide a conduit for advice to providers in community settings, so that patients can receive evidence-informed care close to home and (2) they may provide a referral system that creates access to evidence-based care in the specialty setting. This study investigated these issues using a case study of a network in the Midwest USA that connects an academic medical center (AMC), recognized by the National Cancer Institute for its cancer research and treatment, to community practices.

## Methods

This study sought to understand not only *if* the network created links between settings, but *how*, *when*, *or why* interactions in the network led to evidence-based actions. Case studies can be descriptive of a phenomenon, but they can also be explanatory, seeking the answers to “how” or “why” questions in real-life context, often employing theory in explanation building [33, 34]. We employed an embedded single-case study design, which involves multiple embedded units of analysis within a single case [33]. The rationale for the single-case design is that we sought in-depth understanding within a single network, situated within a common social and organizational context. The use of multiple sub-units (i.e., embedded case units) allowed us to study *how*, *when*, *or why* the network operated effectively in particular patient trajectories, and in doing so, to identify factors related to evidence-based care.

Consistent with case study methodology, there was more than one type of data analysis. Social network analysis (SNA) is the study of connections between actors (people, organizations, etc.) in a network. Within the social sciences, SNA has been applied across various disciplines to everything from the study of disease spread to the dynamics of family interactions. While the objectives of SNA studies in the social sciences are diverse, all have a fundamental interest in the identification or measurement of social networks or relationships of people or organizations. By studying network patterns, we can see which actors are connected or disconnected, as well as how things like information move between actors. For this study, SNA was used to describe which patients and providers were connected, including the type of connection they had, e.g., advice-giving between providers.

Process tracing is a case study method that sequentially traces historical case narratives, including variation, to understand links between causes and outcomes [35]. For this study, it was used to trace the history of the embedded case units and understand how factors in the network led to evidence-based actions. Comparative analysis of the embedded case units and theoretical explanation building (i.e., how does theory help us understand what is happening in the embedded units) were adopted for the purpose of understanding variation. Together, these methods were used to *describe if* the network created links between settings and what kinds of links (SNA), and *explain how*, *when*, *or why* interactions in the network led to evidence-based actions (process tracing).

**Fig. 1:**
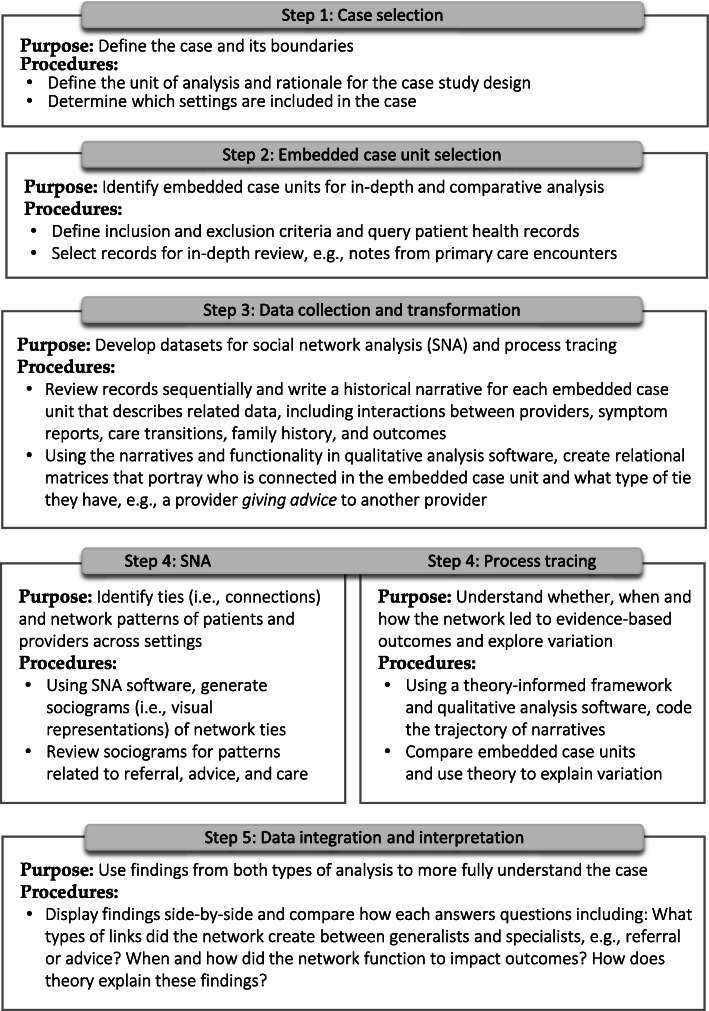
Case study procedures

The study followed five procedural steps, as shown in Fig. 1. Data analysis and interpretation were informed by Everett M. Rogers’ and Mark S. Granovetter’s theories on diffusion of innovations and strength of weak ties, which propose that interpersonal connections (i.e., ties) provide a way to transfer information and potentially persuade people to adopt new practices [36, 37]. When individuals are connected to others who they do not usually interact with (i.e., people with whom they have a *weak tie*), they may gain access to *novel* information. They may also be persuaded to change their practice, for example adopting a new clinical guideline, based on trust in the expertise and qualification of outside experts [36, 37].

Analysis and interpretation were also informed by constructs from relationship-centered care (RCC) [38]. RCC is a theoretical framework from medical education that defines the clinician-clinician relationship as requiring self-awareness, knowledge of other professions, effective communication, and trust, openness, and humility [38]. It has been adapted to the organizational context to highlight qualities that support relationships, including diversity of mental models (capitalizing on different perspectives), trust that others are capable and committed, the use of a mix of “rich” (e.g., in-person and synchronous) and “lean” (e.g., electronic and asynchronous) communication [39]. RCC may help explain the manner in which community providers and AMC specialists maintain their expert positions while developing shared understanding of the best care for the patient.

### Step 1: Case selection

The case was the network, which includes an AMC and community oncology and primary care practices in the upper Midwest. Goals of this particular network include expanding community practice access to AMC expertise through consultation and promoting seamless connections to the AMC when needed. The AMC has been ranked top in the nation in several cancer-related specialties and is considered a high-volume practice in cancers including colon cancer. It is also an NCI-designated national comprehensive cancer center and employs physicians and scientists that participate in guideline-authoring groups including NCCN.

The data were limited to a single disease type in order to study interactions among a small subset of patients (hereinafter referred to as “case units”) and their providers. Young-onset colorectal cancer (CRC) and risk-related conditions was selected as the disease focus based on growing attention to incidence and prevalence [40, 41], the potential to address diagnosis and treatment in the community setting [42, 43], and the availability of specialist expertise and care in the AMC that could benefit community patients and providers via advice or referrals, including experts in young-onset CRC, family cancers, and genetics. Some of these experts are involved in developing evidence-based care pathways for the AMC or are engaged in national cooperative groups that develop evidence-based, consensus-driven recommendations. Community practices are at least 40 miles from the AMC. To understand factors related to differences in setting, as well as differences in training or expertise, we also included primary care practices directly affiliated with the AMC (referred to herein as “academic primary care”). The academic primary care practices are geographically proximal and share an organizational structure with the AMC.

### Step 2: Embedded case unit selection

**Fig. 2:**
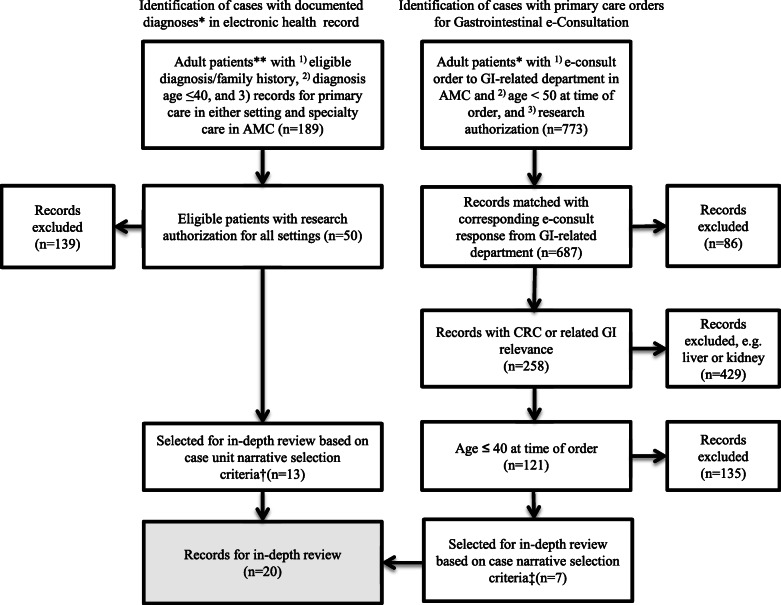
Selection of patients for narratives. * Diagnoses included hereditary colon cancer syndromes (FAP, Lynch, HNPCC), colorectal cancer, or disease related to heightened risk of colorectal cancer. **Adult patients age 18 years or older. † Criteria include (1) primary care records available before diagnosis and (2) ≥ 1 year of records for review (excluding patients presenting only for second opinion or surveillance after outside diagnosis). ‡ Criteria include (1) primary care records available before diagnosis, (2) ≥ 1 year of records for review (excluding patients presenting only for second opinion or surveillance after outside diagnosis), and (3) subsequent diagnosis or GI-related plan of care (excludes orders that resulted in no finding related to GI diagnosis). Abbrev: AMC, academic medical center; GI, gastroenterology

The case units were selected based on patient characteristics. As shown in Fig. 2, patient criteria included (1) age 18 or older; (2) diagnosis at or before age 40 of colorectal cancer, hereditary colon cancer syndromes (FAP, Lynch, HNPCC), or other factors related to heightened CRC risk; and (3) medical records for both community and AMC care or consultation. Age 40 or younger was selected consistent with ages experiencing large increases in CRC incidence and because those patients may be most likely to benefit from expertise related to risk assessment, diagnosis, and care. It is also consistent with the recommendation that family history taking begin by age 40. Patient records were excluded if they did not have research authorization. The subsequent decisions on which records to retain were based on review to determine adequacy of data. Patient units were excluded if (1) there were no primary care records available before diagnosis and (2) there were less than 1 year of records for review. This excluded those patients presenting only for a second opinion and those presenting for disease surveillance after being diagnosed at a facility outside the network. Twenty case units were identified for in-depth review and analysis (11 women and 9 men).

### Step 3: Data collection and transformation

Records from the first entry after January 1, 1990, and on which the patient was at least 18 years of age through December 30, 2018, were extracted for in-depth review. The term “record” in this study refers to clinical documentation and notes, as well as patient forms. The median span of records for the units reviewed was 10 years (range 1.4, 21.1 years). This allowed for tracing histories for documentation of early conversations about family history or genetic testing or counseling referrals. It also allowed for following some patients post-diagnosis and into survivorship, which is a period during which patients may be receiving care in primary and specialty care settings or transitioning between them.

A narrative was composed for each case unit. A member of the study team (JLR) reviewed all records that were associated with a relevant clinical department (e.g., primary care, oncology, medical genetics, gastroenterology), as well as patient forms such as those documenting health history, in order from oldest to most recent and identified text related to the topic of young-onset CRC or risk-related conditions. This included family history and genetics, symptoms, assessment and screening, diagnosis, and treatment or surveillance. The narrative, arranged chronologically, summarized the situation and the actors involved. Examples of narrative passages include the following:

- Patient symptoms and diagnoses, as well as time from first symptoms to diagnosis
- Communication between providers within and across settings including communication mode and whether the communication was related to advice-seeking
- References to clinical guidelines or other evidence sources
- Recommendations for follow-up or referral, as well as the reason for referral
- Notes demonstrating attitudes or beliefs related to cancer risk, diagnosis, or care
- Instances of care coordination or handoffs between providers or settings
- References to barriers or facilitators of patient or provider access (e.g., financial)

Using functionality in NVivo 12 Plus (QSR International), narratives were transformed into relational matrices (i.e., tables of ones indicating a tie and zeros indicating absence of a tie between any two actors in the network) for the SNA. Each tie was coded to show the type of relationship between two actors. These directional tie types, some between patients and providers, and some between providers, included the following: gave eConsult or notes advice to; gave personal advice to; made referral to; provided community setting specialty care for; provided primary care for; provided genetics advice to; and provided academic setting specialty care for. Patient and provider names were removed and replaced with study identifiers (PT=patient and PA=provider). “Community setting specialty care” was provided by oncologists, radiologists, and other specialists who worked in a community setting and generally provided care for patients who lived in the local community. They often served as generalists in their field, e.g., oncologists who treated a range of cancers. In contrast, AMC specialty care was often provided by subspecialists, e.g., CRC oncologists, or other clinicians with expertise typically not available in community settings, e.g., genetics. Definitions of the types of links (advice, referral, and care) and examples are shown in Table 1. Ties were identified as eConsults or notes (i.e., lean communication channels) or personal (i.e., rich communication channels) because diffusion of innovations and RCC stress the important of interpersonal communication and trusting relationships, but strength of weak ties refers to the value of connections between individuals who are not closely associated and do not interact often. Identification of lean and rich ties was aimed at understanding whether the type of tie mattered in this context. The network assembled included 20 patients, 226 providers, and 346 relationships.

**Table 1:** Definitions of link types and examples

Link	Definition	Example scenario
**Advice**	Link between two providers where the purpose is to ask for advice about the best-evidence approach, either using lean or rich communication channels^a^	A community primary care provider submits an eConsult to an expert in the AMC for help interpreting screening guidelines for a young person with a strong family history of colorectal cancer but no other risk factors.
**Referral**	Link between two providers where the specialist referral is the evidence-based approach, or the specialist is engaged to provide evidence-based care	A community-based oncologist refers a patient to the AMC to have a consultation with a specialist in young-onset colorectal cancers or to get treatment only available through a clinical trial at the AMC.
**Care**	Link indicating that the patient is receiving primary care or specialty care^b^	A patient receives primary care from several different clinicians in her local community setting. She also travels to the AMC to have two face-to-face meetings with an expert in family cancers and medical genetics.

^a^In this study, lean communication channels are text-based and asynchronous, while rich communication channels are verbal and synchronous

^b^Care includes in-person visits as well as direct provider-patient communication, e.g., through a patient portal

### Step 4: Data analysis

The relational matrices and narratives served as data sources for SNA and process tracing. The fundamental objective in SNA is the identification or measurement of social networks or ties between people or organizations [44, 45]. Sociograms are visual representations of the individuals in the network and their ties. For this study, analysis began with the generation of a sociogram that showed how all members of the network were connected. This illustrated which actors were more centrally located and more connected, and which were on the periphery and therefore less connected. Next, the researcher generated sociograms displaying the different directional ties (from one actor to another) described above, e.g., made referral to, gave personal advice to. Individuals located along the path between two others were considered to have a central (i.e., strategic) position to influence the flow of advice or referrals [44, 45]. Individual (egocentric) sociograms were also created for each patient, showing the patterns of all the providers involved in their care, including those who were involved only in that they provided advice to a clinician caring for the patient. Egocentric sociograms were subsequently added to narratives to aid in process tracing analysis. Sociograms were created using UCINET 6 and NetDraw (Analytic Technologies).

Process tracing in this study was aimed at understanding when the network led to evidence-based outcomes. Evidence-based medicine (EBM) has been defined as the systematic assessment of evidence (e.g., from syntheses of high-quality randomized clinical trials), along with clinical expertise and patient preferences, to make the best clinical decisions [46–48]. Evidence, often codified in clinical practice guidelines, helps ensure that care does not “vary illogically from clinician to clinician or from place to place” [49]. Cancer clinical guidelines outline best practices such as when patients should receive preventive screening, when healthcare providers should make referrals for suspected cancers, what first-line treatments are recommended based on disease characteristics, and how pain and fatigue can be best managed [50]. Outcomes in this study were defined as evidence-based when documentation referenced clinical guidelines, emerging science from clinical trials, or an expert-informed action or course of care. Our definition of evidence-based did not intentionally omit patient values and preferences, which may have been involved earlier or later in the decision-making process; rather, it is meant to describe the availability of evidence or the process of seeking or understanding it that could be expected through networks.

Analysis was guided by a process tracing framework, displayed in Table 2, which outlines two paths to evidence-based outcomes that may be created through a network such as this one. First, the network may create access to novel information through advice links with experts in the AMC. Second, the network may create access to referrals. While patients may get a referral to the AMC without being a part of the network studied here, benefits of a network in this context are that referrals may be administratively easier (e.g., in terms of pre-approvals) and providers may have access to shared records. The network may also create situations where a referral is coupled with opportunities for advice or other types of personal connections between providers, which would be unlikely without network arrangements.

**Table 2:** Process tracing framework

Network factor	Pathway 1: Advice	Pathway 2: Referral
**Cause**	Provider access to novel information or specialist advice Potential for multidisciplinary interactions	Patient access to specialized treatment or consultation Potential for multidisciplinary interactions
	↓	↓
**Mechanism**	Change in provider knowledge or attitudes related to evidence-based assessment or care Trust in expertise and opportunities to create shared understanding of best-evidence care	Organizational links to referral systems and shared infrastructure, including clinical notes Effective communication and capitalizing on varied expertise
	↓	↓
**Outcome**	Patients receive best-evidence screening, timely diagnosis, and best-evidence treatments close to home	Patients receive best-evidence consultation and treatment and care is coordinated across settings

The pathways were informed by the theoretical perspectives: diffusion of innovations, strength of weak ties, and RCC. In the context of young-onset CRC, community providers may be less familiar with the nuances of screening recommendations for patients under 50 (including knowledge of genetic or familial risk factors). Credible AMC experts, with whom community providers may have only weak links, may be best positioned to provide novel information. Furthermore, the interpersonal nature of connections is posited as being able to change attitudes, e.g., about evidence-based screening.

Relationship-focused theories like RCC also hypothesize that provider-provider relationships—especially hierarchical ones—require self-awareness, trust, openness, and humility [38, 39] and that opportunities to collabore can lead to information sharing and shared mental models [39, 51]. In this study, providers may build a shared understanding of the best-evidence treatment options by valuing the expertise of both parties—including the knowledge of primary care providers (PCPs) who manage overall patient care—through communication and negotiation.

Using NVivo and methods of qualitative content analysis, text from each narrative was coded to a coding structure based on the elements of the process tracing framework, including codes for evidence-based outcomes as defined above. Codes were also created to facilitate queries related to the theoretical perspectives: e.g., attitudes toward innovation/evidence (diffusion of innovations); trust in the expertise of outside experts (strength of weak ties); and mutual respect (RCC). For each narrative, the study team reviewed pathways to an outcome identified as evidence-based (as defined above). They also considered the relationship of actors as portrayed in the sociograms. Between-case analysis was adopted for the purpose of understanding variation among the narratives and identification of factors related to successful network function. Analytic memos were written to summarize reflections.

### Step 5: Data integration and interpretation

Joint displays that portray findings side-by-side provide structure for integration of mixed methods [52]. For this study, the results of SNA and process tracing were considered side-by-side to assess how their unique contributions informed greater understanding and interpretation of the case findings. More specifically, the results were summarized in a matrix to facilitate team discussion and reflection. Matrix rows represented questions from the study, including the following: What types of links did the network create between generalists and specialists, e.g., referral or advice? When and how did the network function in impact outcomes? How does theory explain these findings?

Where process tracing was used to understand potential mechanisms of individual case units (i.e., within-unit analysis), this data integration and between-case interpretation involved identifying more generalized pathways using existing theory as a guide and explanation building for situations where similar situations had different outcomes. The combination of individual unit explanation and generalized theoretical pathways is a strength of combining these methods and is consistent with calls for the use of mixed methods in SNA in order to build better explanations of networks [53]. Analytic memos were written to summarize reflections.

## Results

### SNA results

**Fig. 3:**
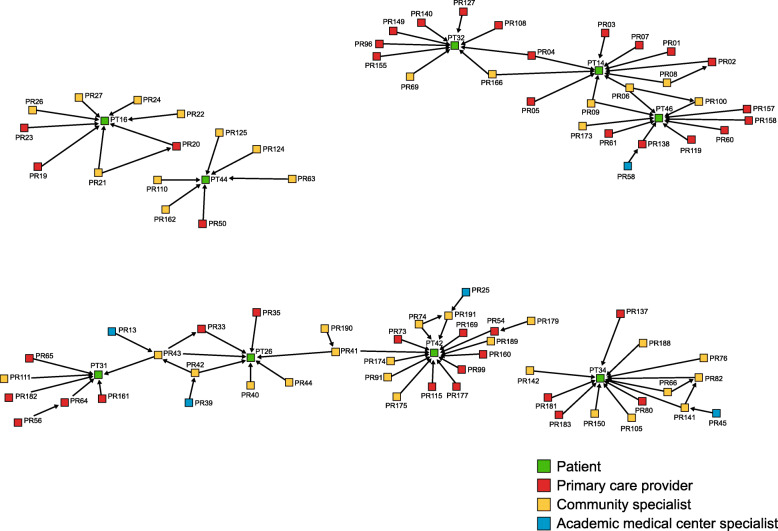
Sociogram of care and advice relationships in the community setting

**Fig. 4:**
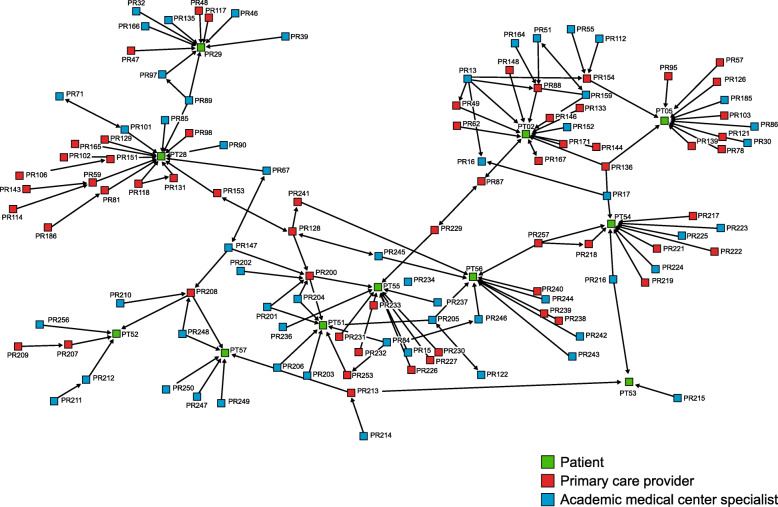
Sociogram of care and advice relationships in the academic setting

Network *advice links* are important because they can diffuse expertise to the community setting, allowing patients to receive best-evidence care close to home. The sociograms showed that the network did diffuse expert AMC advice, but advice connections were fewer in the community setting (Fig. 3) than the academic setting (Fig. 4), as shown by the prevalence of connections from blue to red or yellow nodes. Furthermore, when AMC advice was diffused to the community setting, it typically flowed from the AMC, through a community specialist, and then to the community PCP.

There was also variation in advice modes. In contrast to the academic setting, asynchronous advice (e.g., eConsults or EHR notes) from AMC specialists to community providers was rare, even though technology-mediated advice is a benefit to geographically dispersed networks. More common was synchronous advice (e.g., telephone) from AMC specialists to community specialists, followed by synchronous advice from community specialists to community PCPs.

**Fig. 5:**
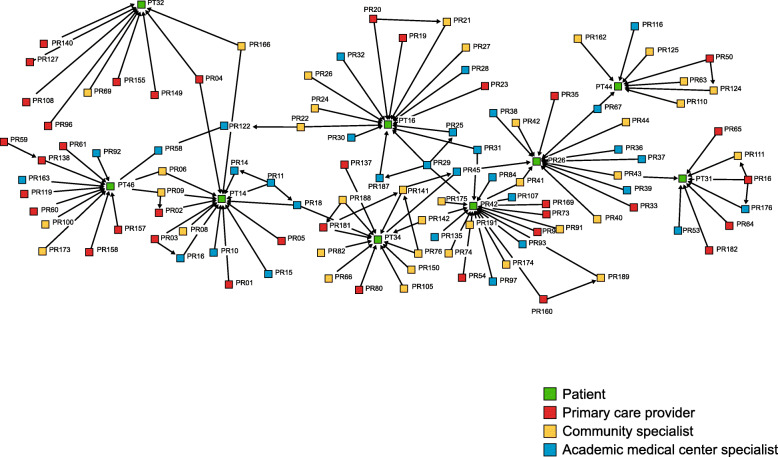
Sociogram of care and referral relationships in the community setting

Network *referral links* are also important because best-evidence care is sometimes to a high-volume or expert setting. This study found that the network opened up access to AMC specialty care for most community patients. Figure 5 includes care and referrals links for patients in community settings. Blue connections represent care that community patients got from AMC specialists. Directional arrows between providers represent provider-provider referrals. They demonstrate that community PCPs made direct referrals to AMC specialists, as well as referrals to community specialists. Referral pathways did not typically go from a community PCP *through a community specialist* on the path to the AMC specialist. This suggests that while community specialists played a key role in advice and knowledge diffusion, referrals sometimes circumvented community specialists on the way to the AMC.

Together, advice and referral findings highlight the issue of individuals’ centrality. Centrality among providers may indicate effective mediation or coordination if they relay information between specialists and patients or coordinate referrals for specialty and follow-up care. On the other hand, central positions may signify gatekeeping if the individual does not facilitate care, advice, or referral but rather interrupts it. Centrally positioned patients may play a unique—and potentially problematic—role if they are in charge of information exchange between providers. While this keeps patients in the loop because information flows through them, it places burdens on patients and minimizes knowledge diffusion between providers, which is a goal of networks.

### Process tracing results

In terms of the first (advice) pathway, process tracing identified variability in PCPs’ documented knowledge of the evidence-based approach (e.g., outlined in clinical guidelines) in situations that involved symptoms like rectal bleeding or risk-based cancer screening, as well as variability in whether or how soon they activated the network for expert advice. When activated, the network served to provide advice on evidence-based care, including guidance on cancer screening recommendations. There were situations where this advice was not only novel but corrected misinformation. This was the situation when a PCP queried advice from an AMC specialist even though he thought he understood the screening guideline; the specialist was able to correct the recommendation by providing additional expert interpretation of the screening guideline. In another example, a PCP sent an eConsult to a specialist because he was confused about how to interpret colonoscopy results, in light of the patient’s family history. The clinical note documented his need for the specialist’s expertise to assist in developing the appropriate care recommendation. In contrast to the limited numbers of situations where community PCPs directly reached out to AMC specialists for advice, community specialists regularly documented consulting AMC specialists for advice.

Process tracing also highlighted the potential of lean communication channels for advice between the AMC and community setting providers. In the academic setting in particular, PCPs used eConsults as an efficient way to get AMC specialist advice. Effective lean communication between and across settings also occurred when providers read each other’s notes. Specialist documentation provided an electronic advice “paper trail” for other specialists or the PCP. For two patients, there was no interaction between the community specialists and the genetics specialist in the AMC, but the community specialists documented awareness of the genetics consult advice via EHR clinical notes. However, there was also evidence in nearly half of the case units that the existence of notes in the EHR was not in and of itself cause for clear communication of advice or closed-loop referrals or follow-through. Many of these failures occurred when providers did not appear to be aware of documentation that may have informed care. Incorrect patient self-reports further obfuscated documentation in the record. Other communication failures were related to difficulty in accessing or summarizing information stored in the record, especially when information was documented in individual visit notes or portal messages with patients.

In terms of the second (referral) pathway, process tracing found that the network provided referral access in 13 of the 20 patient units, based on EHR documentation of referral as the recommended course of care. In two additional case units, the network opened up access through patient self-referral. In most narratives, the referral was for an evidence-based action, e.g., consultation with gastroenterology or medical genetics specialists who informed best-evidence screening recommendations. For those with a CRC diagnosis, it also included best-evidence treatment regimens not available in the community setting and informed by multidisciplinary AMC expertise. The network did not, however, consistently open up access to specialty referrals for all patients, and there is evidence that not all providers used the same threshold for referral (e.g., referral for genetics consultation). Some notes and messages suggest that patients, at times, advocated for referrals. When a referral to AMC specialty did not involve all of the community providers, there was often evidence of confusion about next steps for follow-up.

Communication about follow-up is critical for high-quality, coordinated care, but detailed referral communication can also serve as a method of expert knowledge diffusion. While not the same as the network advice pathway described above, when community providers were exposed to the evidence-based decision process used by AMC experts, e.g., synchronously via real-time access to e-tumor board discussions or asynchronously through clinical notes, it created a potential opportunity for learning. In one narrative, the AMC gastroenterology specialist outlined in her clinical note the four multidisciplinary specialists whose expertise could help determine the best course of care, the reasons for consulting each, and the outcome of these discussions. That clinical note—if read by the referring provider—could inform future patient care in the community setting, even without a direct advice link being activated.

### Mixed methods interpretation

The SNA and process tracing results provided complementary insights into how the network functioned. The SNA showed (dis)connections and highlighted patterns in advice pathways. It also portrayed patterns by communication mode, i.e., whether the advice link used a lean (i.e., electronic) or rich mode of communication. Visual sociograms identified patterns that may not otherwise have been recognized.

The process tracing provided nuanced understanding of conditions when ties were activated, e.g., when a provider documented needing expert advice to interpret a clinical guideline. Furthermore, where SNA identified the central position of specialists in advice links, process tracing added context for whether and when providers or patients were (in)effective knowledge brokers. Process tracing also provided context for different referral decisions, highlighted the role of patient advocacy in some referrals, and illustrated how failure to formalize referrals resulted in fragmented care coordination and confusion about the best evidence course of care. Together, these findings suggest factors related to network success, i.e., factors whose presence or absence appears related to whether the network opened up access to evidence-based care based on advice or referral, as shown in Table 3. However, they also highlight that, along a patient trajectory, there are multiple points at which evidence-based outcomes may be disrupted. For example, in one narrative, the patient was referred to a medical genetics specialist at the AMC because of her strong family history. That referral was the evidence-based approach to risk assessment for someone with her clinical situation. However, subsequent failures in the way the specialist’s screening recommendation was conveyed to the PCP disrupted an evidence-based approach to screening in the community setting.

**Table 3:** Factors related to successful network function

• When community providers recognized the need for AMC advice or referral and knew how to activate the network (directly or through a community specialist) • When opportunities were available for lean and rich communication • When centrally-located providers effectively brokered information between other providers and between other providers and patients • When referrals had adequate information sharing and follow-through • When all providers were formally included in advice and referral links • When providers had time and gave attention to documentation, and when documentation was easy to access or summarize	

## Discussion

Networks are a promising strategy to address community cancer disparities because they provide access to expert advice and care. This study found that an exemplar network created advice and referral opportunities leading to evidence-based care in many but not all situations. However, the significance of this study rests with this variability. In allowing for identification of more and less successful network function, it provides insights into factors that, when addressed, may potentially lead to care that is patient-centered, evidence-based, and coordinated.

Advances in complex fields like genomics, along with the complexities of treating cancer alongside multiple comorbid conditions, pose challenges for community providers [54]. A network benefit is that community providers can go to specialists when they need information or advice, rather than needing to maintain awareness of complex specialty information on their own. While this study identified many advice connections, it also identified variability in whether or when they were activated. Failure to activate network links suggests there are remaining challenges to network awareness or related costs in the time and effort to activate it. Education related to the availability of expertise may be necessary to increase its utilization. Most importantly, development of these relationships requires time that many PCPs may lack.

Networks should also create opportunities for lean communication channels, especially on topics such as risk assessment or recommended screening, where asynchronous advice may be sufficient. However, in addition to lean and asynchronous communication methods, opportunities for in-person or real-time electronic interactions may help build a culture that showcases the diversity of available expertise to network members, e.g., educational seminars or case review sessions. Social networks are in fact critical to the types of interpersonal influence that impact innovation adoption, especially across professional groups. However, these types of personal connections are time and resource intensive, especially in a geographically dispersed and financially lean environment. Assessment of network links also highlighted the role of brokers. Community specialists, in particular, were often able to help broker expertise between AMC specialists and community PCPs. Given the geographic spread of the network, this method of knowledge diffusion from specialist to specialist may be efficient and lessen the burden on PCPs, especially if community specialists know enough to effectively facilitate an appropriate level of information flow.

Problems arose when patients were designated as brokers. Engaging patients in conversations and decisions about their care is critical. However, this study found challenges when patients were put in charge of communication and coordination between their providers. Systems that allow transparency of information flow between all parties—e.g., shared medical records that are available to patients through online patient portals—have potential to ensure all members of the team, including patients, have the same level of information.

Similar to activation of advice connections, providers need to know what types of referrals are available and when they are appropriate, and the network can serve a triage function in this regard. Triage systems such as eConsults can ensure referrals are used appropriately, minimizing burden on the system and patients. A recent study of eConsults in a safety-net system found decreased need for face-to-face consultations and reduced average per patient per month costs with specialty eConsults [55]. Ability to access documentation is critical to lean methods of information sharing. This study found that the network was effective when all providers (and the patient) were aware of decisions. In contrast, communication gaps often resulted in closed loop failures when no one was in charge of ensuring recommended follow-up. Not only did such referrals leave some providers unaware of clinical decisions, they hindered the potential for community providers to gain additional expertise, a benefit of network diffusion.

In this study, network failures occurred when providers did not access documentation that might have informed care, including documented cancer family history. Better genetic family history taking may improve identification of high-risk patients [56], but electronic systems need to support that. EHR-supported family history information/triggers may be especially important in the primary care setting, where change in provider assignment may be common and where providers frequently operate as members of a care team. If lean communication is an important strategy in geographically dispersed networks, electronic systems must make it efficient for providers to access documentation, and providers must have ample time to create the detailed documentation that supports information diffusion.

### Strengths and limitations

This case study design had several strengths including the use of multiple methods to build a more complete picture of the phenomenon, which is consistent with calls for greater use of mixed methods in SNA [53]. Methods of triangulation also provided an opportunity to assess the consistency of findings from the different methods [57]. Use of existing EHR data for the SNA minimized respondent burden and recall bias typically associated with SNA questionnaires. This study was also novel in creating SNA matrices from narratives. This approach provided detailed insights otherwise unavailable in administrative data. Billing data, for example, demonstrate care patterns but omit advice networks or referrals that do not result in a billable visit. Our method also retained the underlying qualitative narrative, so we could return to it when exploring relationships in a sociogram, for example. This type of small, in-depth qualitative SNA may complement the growing number of studies that leverage large datasets for SNA of shared patient care [58–60]. The range of included outcomes was also a strength. Existing network literature tends to focus on discrete evidence-based practices after diagnosis, such as receipt of a particular cancer treatment. However, the trajectory of young-onset CRC provides several points (from risk assessment through survivorship) where access to a network could improve care. Considering a broader range of outcomes allowed understanding in this disease context, which is practically similar to many disease contexts.

There are limitations of this work. It involved a single network, which limits the ability to generalize to other settings. Further research is needed to assess whether there are other important contextual factors that impact network function. Furthermore, the SNA did not include full enumeration of network connections, limiting the ability to generate network statistics that could identify subgroups or characterize network structure. This work also presents the social network as cross-sectional, although actors joined the network at different times. Future longitudinal analysis of a fully enumerated network could provide important insights into how the network has changed over time. Finally, the case unit selection procedures excluded patients who did not receive any care in the AMC, and therefore may have excluded some that could yield rival explanations. Future research could provide insights into case units where patients are completely unconnected to the AMC network.

## Conclusion

These findings suggest that the network created opportunities to reduce cancer disparities in community settings, but development of the network alone did not ensure access to advice or referrals. Full implementation and uptake of the network is necessary to leverage its potential. The identification of factors related to network success in this study provide some evidence for actionable recommendations on how to improve networks so that they serve the purpose of eliminating disparities in community cancer care.

## Data Availability

The full dataset contains identifiable information and is not publicly available due to confidentiality policies.

## Abbreviations

CRC: Colorectal cancer
AMC: Academic medical center
SNA: Social network analysis
